# Immune-mediated glomerular diseases: new basic concepts and clinical implications

**DOI:** 10.1007/s00441-021-03509-5

**Published:** 2021-08-31

**Authors:** Ulf Panzer, Tobias B. Huber

**Affiliations:** 1 III. Department of Medicine, University Medical Center Hamburg-Eppendorf, Martinistr. 52, 20246 Hamburg, Germany; 2 Division of Translational Immunology, III. Department of Medicine, University Medical Center Hamburg-Eppendorf, Hamburg, Germany; 3 Hamburg Center for Translational Immunology (HCTI), University Medical Center Hamburg-Eppendorf, Hamburg, Germany

## Introduction 

Immune-mediated glomerular diseases (Glomerulonephritides, GN) represent a heterogeneous group of disorders. When considered as one disease category, GNs are a major cause of end-stage renal disease worldwide and are associated with significant morbidity and mortality. Most forms of GN are characterized by a pathogenic immune response against renal autoantigens or by manifestations of systemic autoimmunity in the kidney. All forms result in renal tissue injury, which, depending on the immunopathology in the individual patient, can lead to a range of symptoms from heavy proteinuria to rapid loss of renal function. Even though recent years have seen considerable progress in the understanding of the immunopathogenesis of different GN forms, most treatment strategies still consist of corticosteroids and cytotoxic agents, aimed at suppressing the complete immune system. Unfortunately, these nonspecific therapies often bring with them incomplete efficacy and disabling side effects, highlighting the urgent need for more specific and individualized treatment strategies.

In this special issue of CTR “Immune-mediated glomerular diseases: Basic Concepts and Clinical Implications,” we highlight new developments in the field of lymphocyte and chemokine biology in renal autoimmunity, summarize recent advances regarding the interaction of immune cells and renal cells in glomerular diseases, and finally present new technologies and treatment options for this patient group.

## Expanding role of lymphocytes and chemokines in autoimmune kidney diseases

T cells are key drivers of autoimmune diseases, including crescentic GN. Many effector mechanisms employed by T cells to mediate renal damage and repair, such as local cytokine production, depend on their presence at the site of inflammation. In their article “ T helper cell trafficking in renal inflammation” (2021 in this issue) Riedel et al. discuss the current knowledge of mechanisms and functions of T cell migration in renal autoimmune diseases with a special focus on chemokines and their receptors.

While interferon-gamma producing Th1 cells and interleukin-17 secreting Th17 effector T cells are pathogenic drivers of glomerulonephritis, regulatory T cells (Tregs) potently protect from renal tissue injury. Recently, it has become evident that different Treg subtypes exist. In the article “The role of Treg subtypes in glomerulonephritis” (2020 in this issue), Herrnstadt and Steinmetz summarize the currently available data about specialized Treg subsets and discuss their role in GN.

However, there is also evidence for other immune cell populations with immunosuppressive function in renal inflammation. In their review “Immune regulation in renal inflammation” (2021 in this issue), Neumann and Tiegs provide a comprehensive update on immune-regulatory mechanisms of these cell populations, focusing on the function of the CD8 + T cell response and its regulation by co-inhibitory receptors in human and experimental crescentic glomerulonephritis.

In the past, proinflammatory effector T cell subsets were viewed as terminally differentiated lineages with limited flexibility. However, it is now clear that Th17 cells can in fact have a high degree of plasticity and convert, for example, into anti-inflammatory Tr1 cells. In the article “T cell plasticity in renal autoimmune disease” (2021 in this issue), Soukou et al. discussed how targeting Th17 cell plasticity could be envisaged as a new therapeutic approach in patients with glomerulonephritis.

In the article “Regulation and function of CX3CR1 and its ligand CX3CL1 in kidney disease” (2021 in this issue) by von Vietinghoff and Kurts, the important functions of renal dendritic cells in inflammatory kidney disorders are discussed, with a special focus on the regulatory mechanisms of the CX3CL1-CX3CR1 axis in renal inflammation.

## Interaction of the immune system with renal tissue cells

Immune cells actively contribute to tissue damage in immune-mediated GN. In recent years, the interplay with resident kidney cells for the initiation and maintenance of inflammation has become the focus of attention, and in the article “Parietal epithelial cell dysfunction in crescentic glomerulonephritis” (2021 in this issue), Wong et al. focus on the pathophysiological role of parietal epithelial cells as a vital part of crescent formation in glomerulonephritis describing their complex cross-talk with the diverse surrounding cell subsets.

The complement system has long been an underestimated research topic in immune-nephrology, but Zipfel et al. in their article “Complement catalyzing glomerular diseases” (2021 in this issue) show how dysregulated parts of the complement cascade lead to and sustain inflammatory processes in the kidney in primary complement driven diseases and other forms of cGN, suggesting comprehensive complement targeting strategies.

The turnover of intracellular molecules is crucial in homeostasis and, all the more, under inflammatory conditions. In the review “Lysosome function in glomerular health and disease” (2021 in this issue), Meyer-Schwesinger provides the current basis of the lysosome’s role as a key player in the integrity and function of glomerular cell types, thus being in the center of lysosomal storage diseases as well as many forms of glomerulonephritis.

The immune system, and its interaction with the autonomic system, plays a prominent role in the initiation and maintenance of hypertension and significantly contributes to end-organ damage. In their article “Immune mechanisms in arterial hypertension: Recent advances” (2021 in this issue), Wenzel et al. discuss the mounting evidence of both innate and adaptive immune cells’ involvement in the development and consequences of arterial hypertension, with a special focus on the role of high sodium intake as a key driver of this deleterious interplay.

In recent years, huge progress has been made in the understanding of the pathophysiology of membranous nephropathy as one of the leading causes of nephrotic syndrome in adults. In their review “Perspectives in membranous nephropathy” (2021 in this issue), Tomas et al. update the readership on the recent advances made in experimental and clinical research of membranous nephropathy and discuss the potential roles of novel antigens and the deciphering of glomerular injury mechanisms for new pathogenesis-based treatment strategies.

Yet another cause of nephrotic syndrome in adults, (primary) focal segmental glomerulosclerosis, remains at the center of attention. In their article “Immune-mediated entities of (primary) focal segmental glomerulosclerosis” (2021 in this issue), Braun et al. describe the cumulative knowledge of genetics as well as the glomerular and immune cell cross-talk implicated in immune-mediated (primary) focal segmental glomerulosclerosis, fully summarizing the complex interactions of podocytes with soluble factors and leukocytes leading to glomerular scarring.

## New technologies and therapies in kidney immunology and inflammation

Fundamental advances in OMICS technologies, especially in high-dimensional single-cell analyses, have been made in recent years. For the first time, these techniques allow high-resolution analysis of the immune response in minute amounts of biopsy material obtained from inflamed human kidney tissues. In their article “Single cell biology to decode the immune cellular composition of kidney inflammation” (2021 in this issue), Zhao et al. highlight recent advances of single-cell RNA profiling of immune cells isolated from healthy and inflamed kidney tissue with an emphasis on T cell subsets that will help to decipher kidney disease etiologies and mechanisms which will potentially guide future therapeutic interventions.

In the era of an expanding armamentarium of antibody-based treatment approaches, novel strategies for treating immune-mediated kidney disease are eagerly investigated. In their review “Nanobodies: New avenue to treat kidney disease” (2021 in this issue), Wanner et al. comment on the promising results of the use of nanobodies, single-domain antibodies derived from camelid heavy-chain antibodies, as a promising and highly specific new class of biologicals in the treatment of autoimmune kidney diseases.

Alongside new treatment strategies, novel technologies for investigating pathophysiological principles that recapitulate the human in vivo condition more closely are clearly needed. In the article “Kidney organoid systems for studies of immune-mediated kidney diseases: Challenges and opportunities” (2021 in this issue), Stein et al. focus on organoids, a three-dimensional, multicellular culture system, as a state-of-the-art technology for studying immune cell–epithelial cell interactions and for the discovery of pathogenic pathways of immune-mediated kidney diseases.

In a minimum of time, a multi-systems approach has become the state of the art in medical research, and in their review “Perspectives in Systems Nephrology” (2021 in this issue), Lindenmeyer et al. provide an outlook on the possibilities of a comprehensive and integrative approach to kidney disease classification concurrently using multi-omics data, functional clinical information, and histopathological parameters employing contemporary artificial intelligence methods to tackle big data sets.

Over the last years, large-scale single-cell analyses of immune-mediated kidney disease have largely focused on RNA-based technologies. In order to put protein-based analyzes in the spotlight, Demir et al. in their article “Proteolysis and inflammation of the kidney glomerulus” (2021 in this issue) discuss the pathogenic role of proteolytic systems in inflammatory kidney diseases as well as novel diagnostic tools to investigate proteolytic processes directly from kidney tissue samples that might yield information on individual patient prognosis and guide targeted therapies.

In summary, this series of articles outlines how research into immunological mechanisms and immune-epithelial cell interactions at the single-cell level, combined with deep molecular mapping of human kidney tissue and systematic computational modelling of well-characterized glomerulonephritis patient cohorts, could pave the way to patient-, disease-, cell-, and pathway-specific therapies for immune-mediated glomerular diseases.
